# Human breast milk lipoproteins: A preliminary biochemical characterization

**DOI:** 10.1113/EP093757

**Published:** 2026-06-18

**Authors:** Gabriela Arenas, Andrea Morales, Andrea Leiva, Susana Contreras‐Duarte

**Affiliations:** ^1^ PhD Program in Chronic Diseases, Faculty of Science Universidad San Sebastián Santiago Chile; ^2^ School of Medical Technology Faculty of Science Universidad San Sebastián Santiago Chile; ^3^ School of Nutrition and Dietetics Faculty of Rehabilitation Sciences and Life Quality Universidad San Sebastián Santiago Chile

**Keywords:** apolipoprotein AI/ B, human breast milk, lipoproteins

## Abstract

Human breast milk (BM) is an essential fluid for infant development, with a dynamic composition adapted to the infant's needs. It contains bioactive lipids like cholesterol, typically transported by lactosomes, extracellular vesicles, and milk fat globules. However, classic cholesterol‐carrying lipoproteins have not been described in BM. This study included BM from 22 healthy women attending paediatric follow‐up. BM lipoproteins were isolated by ultracentrifugation, and their density was determined by refractometry. Cholesterol and triglyceride levels in whole milk and from BM‐lipoproteins were quantified using commercial kits. The main lipoproteins were quantified by western blot and Luminex assays. Native gel electrophoresis was used to identify lipoprotein migration patterns. BM lipoproteins exhibited densities between 0.999 and 1.043 g/mL, consistent with very low‐density lipoproteins, intermediate‐density lipoproteins, and low‐density lipoproteins (LDLs). No classic high‐density lipoproteins (HDLs) were detected by density, but apolipoproteins AI and B, markers of HDL and LDL, respectively, were detected. BM lipoproteins contained significantly less cholesterol and triglycerides than whole milk and showed migration patterns similar to adult serum HDL and LDL. This study provides the first evidence of classical BM lipoprotein classes, expanding knowledge of lipid transport in BM and suggesting a potential role in neonatal lipid metabolism and cholesterol homeostasis.

## Abstract (highlights)

- **What is the central question of this study?**
  Are lipoproteins present in human breast milk?
- **What is the main finding and its importance?**
  It is demonstrated for the first time that human breast milk contains lipoprotein‐like macromolecules corresponding to VLDL, IDL, LDL and HDL, containing ApoB and ApoAI main lipoprotein proteins. This expands current understanding of lipid transport mechanisms in breast milk and suggests potential physiological roles in infant lipid metabolism.

## 1 INTRODUCTION

Human breast milk (BM) is a complex and heterogeneous biological fluid necessary for growth and correct offspring development and the primary source of nutrition for the newborn (Eisha et al., 2022; Wijenayake et al., 2023). The World Health Organization (WHO) recommends exclusively breastfeeding for the first 6 months of an infant's life to receive the optimal benefits of maternal milk (WHO, 2026). In humans, BM is rich in nutritious and non‐nutritive bioactive components, including proteins and lipids such as phospholipids, cholesterol and triglycerides (Eisha et al., 2022). BM lipids are the main source of energy (Koletzko, 2017) and also provide essential fatty acids that are critical to an infant's neurodevelopment (Chiurazzi et al., 2021), growth and lipid metabolism, among other functions (Koletzko, 2017). BM lipid transport occurs in diverse ways through small structures called lactosomes, which are 25–30 nm in size and do not contain triglycerides (Argov‐Argaman et al., 2010). Their function is not related to the nutrition of the neonate (Argov‐Argaman et al., 2010) but to early innate and adaptive immune responses (Maheshwari et al., 2024). Human BM also contains other extracellular lipid‐enclosed structures, such as extracellular vesicles and casein micelles, both ranging from 100 to 200 nm in diameter (Hu et al., 2021; Maheshwari et al., 2024; Thum et al., 2023). Extracellular vesicles have roles related to communication between cells (Ginini et al., 2022), while caseins supply the newborn with proteins and ions such as phosphates and calcium (Müller‐Buschbaum et al., 2007). There is also a fourth way of secreting lipids, which is the most classic way to transport lipids in BM: lipids are transported across the apical plasma membrane of luminal cells of the mammary gland into the mammary alveoli. These lipids are packed inside structures containing cytoplasmic lipid droplets (CLD), organelle‐like structures composed mainly of triglycerides surrounded by phospholipid (PL) monolayer and specific attached proteins (Ohsaki et al., 2009). Fusion of the CLDs generates membrane‐bound products called milk fat globules (MFG) (McManaman, 2014), with a specific refractive index of 1.462 (Goff et al., n.d.) and diameter between 0.1 and 15 µm (Maheshwari et al., 2024; Raz et al., 2022). These structures have a role in nutrition (Argov‐Argaman et al., 2010). MFG phospholipids are described as having a structural role in organelles and the plasma membranes (Anna Garczewska‐Murzyn & Michał Smoczyński, 2021), and MFG fatty acids from triglycerides promote healthy neurodevelopment and immune functions (Koletzko, 2017).

Cholesterol is transported in MFGs and incorpored into cell membranes (Yang et al., 2018) and the myelin of the nervous system during the infant's brain growth (Yang et al., 2021). In addition, cholesterol is the substrate for the synthesis of bile acids, vitamin D, hormones and oxysterols that modulate cholesterol metabolism, lipid and lipoprotein homeostasis (Koletzko, 2017; Mutemberezi et al., 2016). BM provides the infant with esterified and free cholesterol obtained from maternal blood located on the basolateral side of mammary alveolar cells and secreted into the alveoli by three proposed pathways (Ontsouka & Albrecht, 2014). One is by MFG transport, the second is passive/carrier‐mediated, and the third is by binding to apolipoprotein AI, where apolipoprotein AI is the main protein present in high‐density lipoproteins (HDLs) (Jomard & Osto, 2020). However, no studies have investigated the presence of HDL in BM.

Interestingly, the delivery of BM cholesterol with breastfeeding has been associated with higher plasma concentrations of total cholesterol (TC) and low‐density lipoprotein (LDL) in breastfed infants than in those fed formula (Shamir et al., 2003). However, to our knowledge, the classic macromolecular complexes, lipoproteins, that transport cholesterol and triglycerides packed in a monolayer of phospholipids have not been described in BM. Consequently, this article aims to determine whether BM has lipoproteins and if so to characterize them.

## 2 METHODS

### 2.1 Ethical approval

All procedures were conducted in accordance with the *Declaration of Helsinki*. The study was approved by the Ethics Committee of the Central Metropolitan Health Service in January 2022. Informed consent was obtained for each participant (Grant ‘Subvención para la Instalación en la Academia’ SA77210098).

### 2.2 Study groups

Women who take their infants for a paediatric follow‐up consultation at the Family Health Center 1, Santiago, Chile were invited to participate in this study (*n* = 22), from whom mature BM (from 1–12 months after childbirth), was obtained to characterize the presence of BM lipoproteins. Clinical information about the women was given by the clinical staff. Healthy women, breastfeeding and over 18 years of age were included in this study. Women who presented pregnancy pathologies such as preeclampsia, gestational diabetes, diabetes mellitus type II, hypertension and thyroid pathologies, among other metabolic pathologies, were excluded from this study.

### 2.3 Lipid profile quantification

A 400 µL sample of brachial venous blood was taken from nursing women (without fasting) for determination of TC, HDL, LDL, very low‐density lipoprotein (VLDL), and triglyceride (TG) levels. TC, HDL and TG were determined via standard enzymatic–colorimetric assays (Cobas Integra Cholesterol (CHOLL), Cobas Integra HDL cholesterol (HDL‐C), and Cobas Integra Triglycerides (TRICL) kits, Roche Diagnostic Corporation, Indianapolis, IN, USA) in a Cobas 8000 series modular analyser (Roche Diagnostic Corporation) at the Clinical Laboratory of the Hospital Clínico UC‐Christus. LDL and VLDL cholesterol were calculated from TC, HDL and TG concentrations by applying Friedewald's equation.

### 2.4 Lipoprotein isolation

BM samples were defrosted and warmed up to 37°C. Lipoproteins from 1.4 mL of BM were isolated by ultracentrifugation as described for serum (Fuenzalida et al., 2020). Briefly, EDTA (10 mmol/L, pH 7.4), aprotinin (2 µg/mL), and phenylmethylsulfonyl fluoride (PMSF; 1 mmol/L) were added to the human BM. The density of the sample was adjusted to 1.24 g/mL with potassium bromide (KBr), and a gradient was generated by adding phosphate‐buffered saline (PBS; 3 mL, density: 1 g/mL). Gradients were ultracentrifuged at 280,000 RCF, at room temperature, for 4 h in a swinging bucket rotor (TST 60.4, Combi, Sorvall Ultracentrifuge, Thermo Fisher Scientific, Waltham, MA, USA). After the ultracentrifugation, the rings obtained were taken from the ultracentrifugation tube one by one with a syringe and stored in an Eppendorf tube. Fetal and adult serum or plasma samples were included as reference controls for lipoprotein characterization and electrophoretic mobility comparison.

### 2.5 Lipoprotein density measurement

After ultracentrifugation, fractions of 200 µL were collected from each sample, and density was determined by refractometry (Spectronic Instruments, NY, USA). Isolated fractions with similar density values were dialysed together in saline solution (mmol/L 150 NaCl, 0.34 EDTA, pH 7.4, 4°C, 48 h) and later stored at 4°C in tubes saturated with nitrogen to avoid lipoprotein oxidation.

### 2.6 Quantification of TC and triglycerides in BM and isolated lipoproteins

TC and TG levels were determined in 10 µL of BM or pooled lipoproteins using assay kits according to the manufacturer's instructions (Spinreact, Barcelona, Spain). The absorbance (505 nm) was quantified using an enzymatic colorimetric reaction. Absorbance was measured with a Synergy HT microplate reader (BioTek Instruments, VT, USA). Results were expressed as mg/dL according to calibration curves (0–400 mg/dL).

### 2.7 Luminex quantification of apolipoprotein AI and apolipoprotein B

Whole BM samples of 10 µL chosen randomly were diluted twice in the assay buffer provided by the kit. The quantification of apolipoprotein AI and B was performed using the Merck kit APOMAG‐62K Milliplex map (Merck, Darmstadt, Germany), according to the manufacturer's instructions.

### 2.8 Protein quantification

Isolated fractions of BM lipoproteins (10 µL) were used to determine protein content using the bicinchoninic acid (BCA) protein assay kit (Thermo Fisher Scientific), following the manufacturer's instructions. A BSA standard calibration curve was used to interpolate the data (0–2000 µg/mL, Sigma‐Aldrich, St Louis, MO, USA).

### 2.9 Western blot

Proteins from isolated BM lipoproteins (150 µg) were separated by 4–15% gradient PAGE in denaturing conditions. Next, proteins were transferred to polyvinylidene difluoride (PVDF) membranes (Thermo Fisher Scientific) for 5 h at 70 V. Later, PVDF membranes were blocked with a solution of 0.1% Tris buffered saline (TBS)–Tween‐20 and 5% milk as the antibody dilution buffer and then incubated with primary mouse polyclonal anti‐apolipoprotein B (ApoB) (1:1000, 4°C, Santa Cruz Biotechnology, Dallas, TX, USA), anti‐apolipoprotein AI (ApoAI) (1:1000, 4°C, Santa Cruz Biotechnology) antibodies. Following the incubation, membranes were washed three times with TBS–Tween‐20 and incubated with a secondary antibody conjugated with horseradish peroxidase goat anti‐mouse antibody (Thermo Fisher Scientific, USA) for 1 h at room temperature. Afterward, the membranes were washed three times with TBS–Tween‐20, and proteins were detected by enhanced chemiluminescence (Supersignal West Femto, Thermo Fisher Scientific, USA) in an image analyser (ImageQuant LAS 500, Thermo Fisher Scientific). The images were quantified with the software ImageJ (NIH, Bethesda, MD, USA).

### 2.10 Polyacrylamide gradient native gel electrophoresis

For polyacrylamide gradient native gel electrophoresis, a protocol was modified from Blom et al. (2003), and polyacrylamide gradient mini gels (3–20%) were used. The separator gel was prepared in a gradient maker with pH 8.8 solution. The stacking gel was prepared with 3% acrylamide at pH 6.8. The samples were previously stained as described. Briefly, 50 µL of BM samples were incubated with 50 µL of lipid stain (1% w/v Sudan Black in ethylene glycol) overnight at 4°C, and then the complete volume was loaded in the gel. The gel was run for 7–10 h at 130 V constant at 4°C in a Tris–glycine buffer, pH 8.3. The gel was subsequently photographed without any further processing.

### 2.11 Statistical analysis

Maternal clinical data are shown as means ± SD. In all the experiments, *n* indicates the number of women or BM samples used. Data were analysed by using GraphPad Prism 9.5.0 software (GraphPad Software, Boston, MA, USA).

## 3 RESULTS

### 3.1 Clinical characteristics of the studied groups

Data were analysed using information obtained from clinical records from the Family Health Centre 1 (Table 1). Variables such as age, height, weight, body mass index (BMI), blood pressure, lipid profile, infant age, gestational age and parity were obtained. The population was mainly overweight, with the arterial pressure of the women being below the normal range (Kumar et al., 2022), and the lipid levels were also below the normal range (Jimenez et al., 1988).

**TABLE 1:** Clinical characteristics of women and the age of their infants.

Maternal and infant variables	Mean ± SD (range)
Age (years)	28.1 ± 1.5 (18–43)
Height (m)	1.6 ± 0.01 (1.47–1.71)
Weight (kg)	73.7 ± 4.0 (44.8–101)
BMI (kg/m^2^)	29.5 ± 1.4 (20.7–38.3)
Diastolic blood pressure (mmHg)	71.2 ± 4.9 (46–97)
Systolic blood pressure (mmHg)	113.2 ± 5.2 (97–140)
Total cholesterol (mg/dL)	188 ± 9.5 (137–264)
Triglycerides (mg/dL)	112 ± 18 (60–320)
Low‐density lipoprotein (mg/dL)	101 ± 8.9 (59–182
High‐density lipoprotein (mg/dL)	60 ± 3.7 (33–92)
Very low‐density lipoprotein (mg/dL)	31 ± 6.1 (10–88)
Parity (number of children)	2.3 ± 0.4 (1–5)
Infant age (months)	4.3 ± 0.8 (1–12)

*Note*: Clinical variables of the dyad. The number of the recruited dyads was 22. BMI: body mass index.

### 3.2 BM lipoprotein isolation and characterization

**FIGURE 1:**
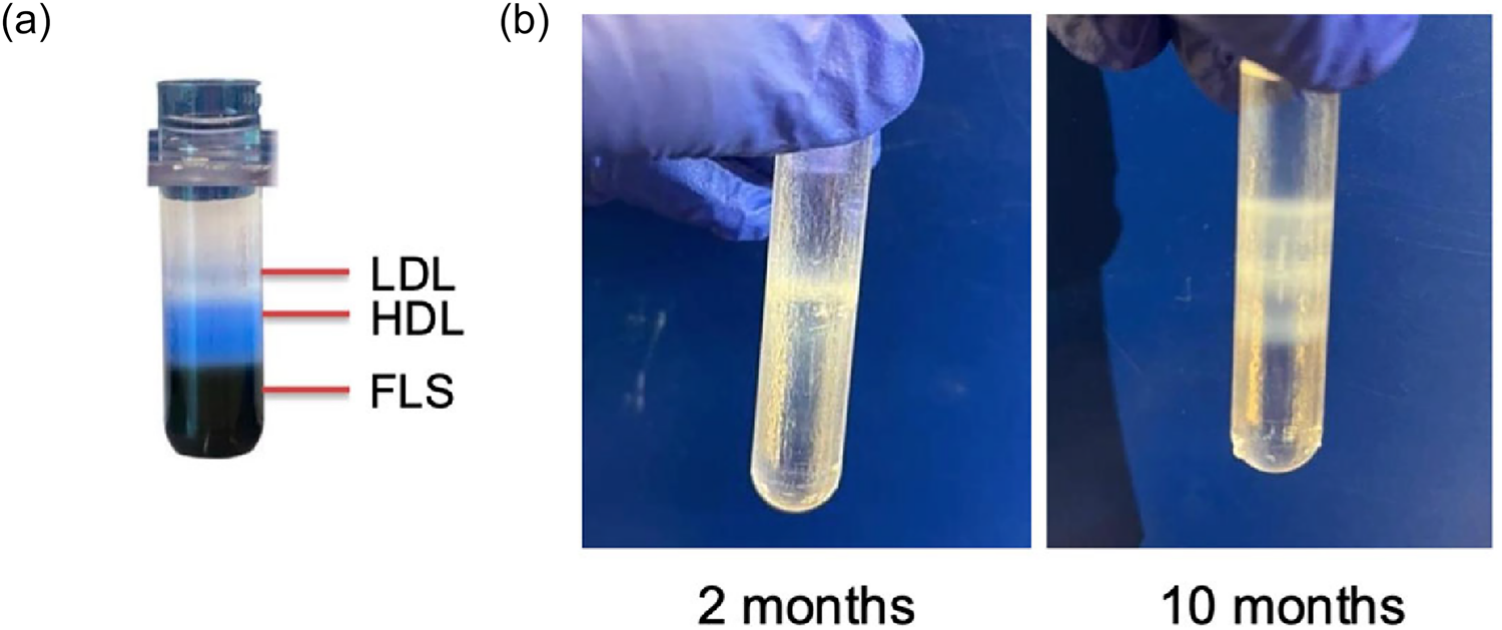
Isolated breast milk lipoprotein‐like macromolecules. (a) Fetal serum lipoproteins. (b) Representative photograph of lipoproteins isolation from 2‐ and 10‐month breast milk. Experiments were repeated three independent times. FLS, free lipoprotein serum; HDL, high‐density lipoprotein; LDL, low‐density lipoprotein.

As shown in Figure 1, macromolecules with distribution patterns similar to lipoproteins were obtained from human BM, from women who were lactating between 1 and 12 months after giving birth (lipoprotein‐like macromolecules). The experiments were repeated three independent times with six different samples. The number of rings corresponding to lipoprotein‐like macromolecules in these BM particles differed according to their lactation stage. Density was measured in the different fractions to evaluate if the rings corresponded to lipoproteins (Table 2).

**TABLE 2:** Density range of 1–10 months of breast milk particles obtained from ultracentrifuged fractions.

	Samples																					
Density/Time	1 month					2 months				3 months						4 months		5 months			10 months	
D1	1.003	1.001	1.002	1.003	1.004	1.000	0.999	1.001	1.003	1.003	1.001	1.003	1.002	1.003	1.000	1.000	1.000	1.004	1.004	1.006	0.999	1.001
D2	1.003	1.001	1.002	1.003	1.004	1.001	1.000	1.001	1.003	1.003	1.002	1.003	1.002	1.003	1.001	1.000	1.001	1.004	1.004	1.006	0.999	1.001
D3	1.004	1.001	1.003	1.003	1.004	1.001	0.999	1.001	1.004	1.003	1.002	1.003	1.002	1.003	1.001	1.001	1.001	1.004	1.004	1.006	0.999	1.001
D4	1.004	1.001	1.003	1.003	1.004	1.001	0.999	1.003	1.004	1.004	1.002	1.004	1.002	1.003	1.001	1.001	1.001	1.004	1.005	1.006	0.999	1.002
D5	1.004	1.001	1.004	1.003	1.004	1.003	1.000	1.007	1.004	1.004	1.003	1.004	1.003	1.004	1.001	1.001	1.001	1.005	1.005	1.006	1.000	1.004
D6	1.006	1.001	1.005	1.004	1.005	1.002	1.003	1.008	1.007	1.007	1.003	1.007	1.004	1.004	1.001	1.001	1.002	1.006	1.005	1.007	1.000	1.006
D7	1.007	1.002	1.006	1.005	1.006	1.003	1.003	1.011	1.008	1.009	1.004	1.007	1.004	1.007	1.003	1.003	1.002	1.007	1.005	1.007	1.001	1.010
D8	1.009	1.004	1.009	1.006	1.007	1.007	1.003	1.024	1.013	1.011	1.007	1.015	1.006	1.008	1.005	1.004	1.004	1.008	1.008	1.007	1.003	1.017
D9	1.011	1.007	1.011	1.008	1.009	1.007	1.006	1.024	1.018	1.015	1.010	1.013	1.007	1.010	1.009	1.002	1.004	1.009	1.008	1.009	1.005	1.019
D10	1.016	1.007	1.015	1.010	1.013	1.011	1.008	1.034	1.022	1.018	1.016	1.022	1.009	1.015	1.010	1.008	1.010	1.011	1.010	1.010	1.008	1.029
D11	1.018	1.007	1.022	1.015	1.015	1.019	1.008	1.037	1.025	1.019	1.025	1.028	1.016	1.020	1.016	1.011	1.018	1.015	1.012	1.013	1.010	1.033
D12	1.025	1.014	1.028	1.020	1.019	1.026	1.022	1.040	1.034	1.028	1.033	1.028	1.027	1.024	1.023	1.023	1.022	1.019	1.015	1.013	1.016	1.037
D13	1.030	1.020	1.035	1.030	1.024	1.028	1.022	1.036	1.040	1.032	1.037	1.037	1.025	1.031	1.034	1.034	1.030	1.022	1.021	1.018	1.019	1.041
D14	1.028	1.023	1.033	1.025	1.028	1.022	1.020	1.450	1.040	1.037	1.042	1.040	1.023	1.034	1.030	1.025	1.034	1.023	1.023	1.023	1.028	1.043
D15	1.034	1.031	1.030	1.032	1.028	1.034	1.030	1.031	1.040	1.044	1.046	1.046	1.036	1.039	1.041	1.030	1.036	1.028	1.022	1.024	1.030	1.029
D16		1.037		1.037		1.033	1.033			1.036	1.037	1.040	1.028	1.041	1.043	1.034	1.030		1.032	1.028	1.033	
D17		1.033		1.039		1.018	1.043				1.020		1.039	1.041	1.042	1.038	1.039		1.022		1.037	
D18		1.037		1.033			1.040						1.039		1.029	1.034	1.022				1.033	
D19		1.038					1.040						1.023			1.016					1.037	
**Mean**	1.013	1.014	1.014	1.016	1.012	1.013	1.015	1.047	1.018	1.017	1.017	1.019	1.016	1.017	1.016	1.014	1.014	1.011	1.012	1.012	1.013	1.018
**SD**	0.011	0.015	0.012	0.014	0.009	0.012	0.016	0.112	0.015	0.014	0.016	0.016	0.014	0.015	0.016	0.014	0.014	0.008	0.009	0.007	0.015	0.016

*Note*: The coloured pattern of the columns corresponds to classical densities for the different lipoproteins: yellow, VLDL; light blue, IDL; green, LDL (Feingold & Grunfeld, 2024). Each column corresponds to 22 different breast milk samples from a particular period of the breast milk isolation. Abbreviation: D, density.

Since classical lipoproteins from serum are categorized according to their density, this parameter was also measured in all the isolated BM lipoprotein‐like macromolecules, as mentioned in Methods. The density of fractions obtained from BM ranged between 0.999 and 1.043 g/mL, which classically corresponds to VLDL, intermediate‐density lipoprotein (IDL) and LDL (Feingold & Grunfeld, 2024). Table 2 also shows in detail the densities obtained from all the samples we used for this study.

### 3.3 BM apolipoprotein presence and quantification in ultracentrifuged particles

After ultracentrifugation, lipoprotein‐like rings were observed, varying from one to three among samples. Their density was within the expected range for plasma lipoproteins. To confirm particle identity, we quantified key apolipoproteins: apolipoprotein AI (ApoAI), the principal HDL protein, and apolipoprotein B (ApoB), the major protein of non‐HDL lipoproteins (VLDL, IDL and LDL) (Feingold & Grunfeld, 2024). Although the classical HDL density range (1.063–1.21 g/mL) was not detected, ApoAI remained present.

**FIGURE 2:**
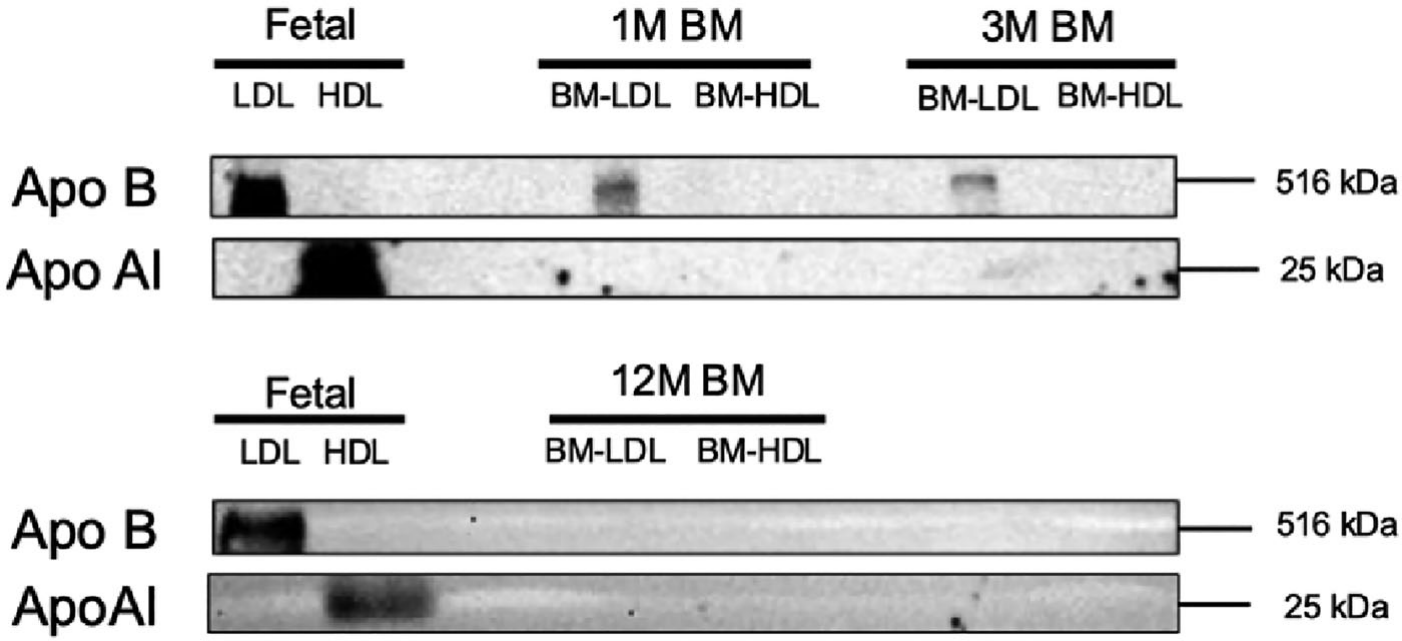
The abundance of apolipoproteins AI and B in breast milk lipoprotein‐like macromolecules. Fetal lipoproteins were used as a positive control for the experiment. At 1, 3 and 12 months, samples of breast milk lipoproteins were determined for ApoAI and ApoB. Cropped western blot images are displayed. ApoAI, apolipoprotein AI; ApoB, apolipoprotein B; BM, breast milk; HDL, high‐density lipoprotein; LDL, low‐density lipoprotein; M, month.

As observed in Figure 2, BM samples from months 1, 3 and 12 were used to perform these assays. Three independent experiments were performed to describe whether some of these apolipoproteins appear or disappear according to the age of the BM. As observed in the representative western blot images for apolipoproteins, ApoB is present at months 1 and 3, and its abundance is not observed at month 12. For ApoAI, it is barely seen at 3 months.

Since ApoAI seemed to be present in 3‐month samples, a more sensitive test was performed to corroborate its presence. ApoB was also measured to confirm its presence. As Table 3 shows, ApoAI and ApoB were present in BM samples.

**TABLE 3:** Quantification of apolipoprotein AI and apolipoprotein B in whole breast milk samples.

BM sample	ApoAI (ng/mL)	ApoB (ng/mL)
Sample 1	3608	597.3
Sample 2	330.8	1127.1
Sample 3	202.4	929.3
Sample 4	77.18	806.0
Sample 5	6368	2768

*Notes*: Five out of the total 22 samples were selected for performing this experiment.

Abbreviations: ApoAI, apolipoprotein AI; ApoB, apolipoprotein B; BM, breast milk.

In addition to density, lipoprotein's main lipids, such as TC and triglycerides, were measured. In this case, whole BM (i.e., with fat) was measured with lipids obtained from pooled lipoprotein‐like macromolecules isolated by ultracentrifugation. As seen in Table 4, macromolecules obtained from BM had 265 and 28.5 times less TC and triglycerides levels, respectively, than whole BM.

**TABLE 4:** Quantification of total cholesterol and triglycerides of whole breast milk and breast milk lipoprotein samples from 2 to 12 months.

Lipid levels (mg/dL)	Mean ± SD	Number of samples
Whole BM total cholesterol	13 ± 47.4	14
Whole BM triglycerides	1653 ± 1191	17
BM lipoprotein‐like macromolecules total cholesterol	9.8 ± 10.9	10
BM lipoprotein‐like macromolecules triglycerides	58 ± 63.4	12

Abbreviation: BM, breast milk.

### 3.4 BM ultracentrifuged isolated particles and their migration pattern

**FIGURE 3:**
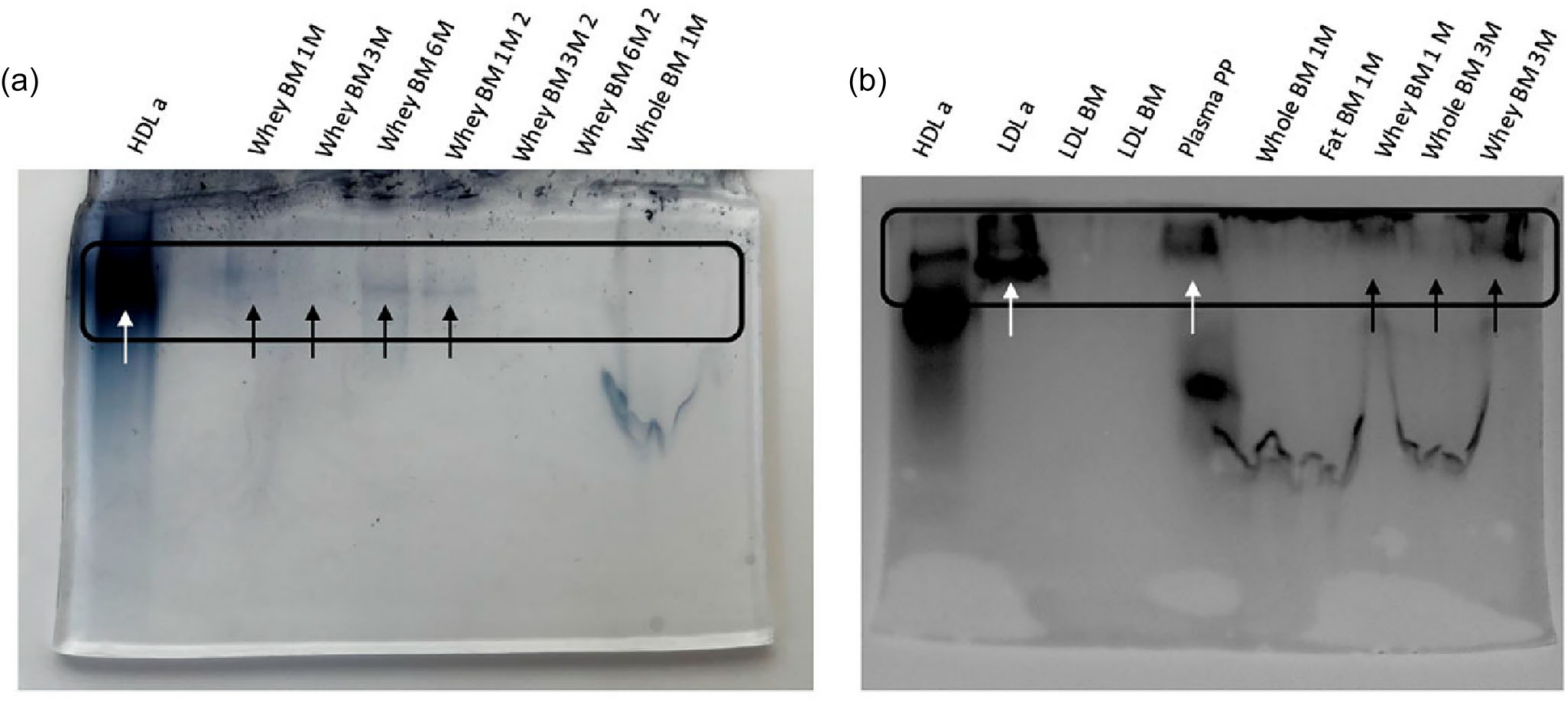
Breast milk lipoprotein migration. (a) Native gel to identify HDL lipoproteins present in breast milk. (b) Native gel to identify LDL lipoproteins present in breast milk. Breast milk samples come from a different donor. The boxes show the migration patterns of each lipoprotein (HDL and LDL). White arrows correspond to control samples, and black arrows correspond to whey breast milk samples. 1M, 3M, 6M, 1, 3, 6 skimmed milk (whey) months of sample age; HDLa/LDLa: HDL or LDL plasma samples from adults; M, month; Plasma PP, plasma sample from women in postpartum period.

To confirm that the isolated macromolecules are lipoproteins, native electrophoresis was performed using adult serum lipoproteins as positive controls (HDLa, LDLa) isolated by ultracentrifugation as well. As observed in Figure 3a, whey from BM (skimmed milk) from 1 month and one sample of 6 months migrate similarly to HDL from adult in the native gels, while in the whole milk, the band is not observed. As observed in Figure 3b, LDL from adults is present at the top of the gel, and so is the LDL from BM, and in the whey of BM from 1 month and one sample of 3 months, with greater abundance.

## 4 DISCUSSION

To our knowledge, this is the first report identifying lipoprotein in human BM. In this paper, we describe the lipoprotein‐like macromolecules whose density corresponds to that of classical plasma lipoproteins. These particles also contain the major structural apolipoproteins, such as ApoAI and ApoB, and possess both cholesterol and triglycerides, supporting their classification as genuine lipoprotein‐like entities present during the lactation periods analysed. This finding extends the current understanding of BM composition and raises important questions regarding their role in infant lipid metabolism, their impact on infant nutrition and their impact on infant health.

Previous work by Gómez‐Gallego et al. (2018) suggested the presence of lipid‐ and protein‐transporting particles in BM. Their study aimed to determine the influence of geography and delivery mode on the milk microbiome. They performed nuclear magnetic resonance (NMR) spectroscopy to characterize the metabolic profile of BM. Particles they named LDL‐ and VLDL‐like in BM, with NMR resonances, structures and mobilities similar to those of plasma LDL and VLDL, were found. Our study expands upon this pioneering observation by employing ultracentrifugation, the gold standard technique for lipoprotein isolation, together with classical density‐based criteria (Feingold & Grunfeld, 2024) to confirm and characterize distinct lipoprotein classes within human BM.

In our study, density was measured and used as a criterion for classifying classical lipoproteins according to the established definitions (Chandalia & Abate, 2004). We confirmed the presence of VLDL, IDL and LDL in BM, along with their major structural apolipoprotein, apolipoprotein B. The electrophoretic mobility observed in the native gel was consistent with canonical plasma lipoproteins. In contrast, no band corresponding to HDL‐like was detected. To assess whether this absence reflected a low abundance or a true lack of HDL particles, density measurements were repeated on the same samples. The physiological presence of these lipoprotein classes in BM might be related to the lipid requirements of the infant, whose circulating lipoproteins are predominantly HDL (Morillas et al., 1992). It is therefore plausible that BM VLDL, IDL and LDL complement the infant endogenous lipid pool, providing an additional source of triglycerides and cholesterol essential for growth. Thus, the detection of these lipoproteins in our samples may reflect a physiological adaptation that supports rapid weight gain during early life. Nevertheless, this hypothesis needs further experimental validation. Moreover, the heterogeneity observed in the rings obtained from the ultracentrifugation experiments (Figure 1) may also reflect dynamic modulation of lipoprotein secretion in response to the infant's metabolic demands as an adaptive process. Interestingly, we observed differences in BM lipoprotein‐like profiles across the lactational ages analysed (1, 3 and 6 months). These temporal variations may reflect physiological adaptations of BM composition to the changing metabolic requirements of the growing infant. During early life, rapid growth and organ development require a high lipid supply, not only as an energy source but also for membrane synthesis and neurodevelopment (Najdi et al., 2025). As lactation progresses, both infant metabolism and feeding patterns evolve, which may influence the secretion and composition of lipid‐transporting particles in BM. Therefore, the differences observed between early and later lactation stages may represent a dynamic mechanism by which maternal milk adjusts lipid delivery to the developmental needs of the infant.

Similar to the progressive rise across lactation of the content of milk fat (Thum et al., 2022), the principal macronutrient used for lipoprotein assembly (Ha & Bhagavan, 2023), the composition of BM lipoproteins likely mirrors the infant's evolving metabolic needs. In support of this interpretation, trace amounts of HDL‐like particles – indicated by the presence of ApoAI detected by western blot and Luminex – suggest a minor but detectable HDL component relative to maternal plasma levels. Since no particles with a density comparable to classical HDL were detected in BM, we hypothesize that HDL‐like macromolecules in this biofluid may differ in density from canonical HDL. These macromolecules are unlikely to be lactosomes because even if they share ApoAI, these particles do not have triglycerides (Argov‐Argaman et al., 2010). Neither are they MFG because the density of these macromolecules is higher. This distinction warrants confirmation in future studies. The discrepancy between our findings and those reported by Gomez‐Gallego et al. may be attributable to differences in sample age: our analysis included BM from 1 to 12 months of lactation, whereas Gomez‐Gallego used only 1‐month BM samples. Additionally, the Luminex assay's higher analytical sensitivity may have enabled the detection of ApoAI in our samples (Gómez‐Gallego et al., 2018). In addition, when the electrophoretic mobility was assessed under native conditions, the BM‐HDL‐like fraction migrated similarly to adult HDL (see Figure 3), suggesting that BM contains macromolecules of similar characteristics, suggesting the presence of structurally related plasma HDL, albeit at low abundance in BM.

The average TC concentration in whole BM was 13 mg/dL in our investigation, with a mean value of 16 mg/dL reported in the literature (Zhang et al., 2022). BM cholesterol levels exhibit natural variability, typically ranging from 9 to 15 mg/dL (Koletzko, 2017), depending on ethnicities, geographic regions and maternal diets (Yang et al., 2021). Accordingly, the cholesterol levels are from 10 to 23 mg/dL in Australia, Europe and the USA (Álvarez‐Sala et al., 2015; Kamelska et al., 2012). Thus, BM cholesterol levels measured here fall within the physiological range described for BM.

BM triglyceride concentrations are known to vary widely and are influenced by maternal diet and the time of sample collection (Ward et al., 2021). In our study, the mean triglyceride concentration was 1.65 g/dL. Previous reports describe concentrations ranging from 3.05 ± 0.39 to 13.8 ± 0.39 g/dL in women consuming diets rich in fat and sugar, respectively (Ward et al., 2021). On a global scale, average BM fat content is approximately 3.4 g/dL (Zhang et al., 2022). We propose that the lower triglyceride levels in our samples may reflect incomplete milk extraction during collection. Indeed, it has been reported that fat content which is 98% triglycerides in BM (Demmelmair & Koletzko, 2018) is almost double in hindmilk (Ramiro‐Cortijo et al., 2020), obtained at the end of a feeding or expression cycle, compared with the foremilk collected earlier (Nielsen et al., 2017). Therefore, if the breast is not completely emptied, the resulting sample will exhibit a reduced fat content.

Regarding the lipid composition of the isolated BM lipoprotein‐like fractions, plasma lipoproteins differ in their cholesterol‐to‐triglyceride ratios, which underlie their classification into distinct lipoprotein types (Kontush & Chapman, 2006). Although we identified both cholesterol and triglycerides in BM lipoprotein‐like particles, the relative abundance and distribution of these lipids in this biofluid remain to be determined. Finally, to assess the size and distribution of BM LDL and HDL‐like, non‐denaturing polyacrylamide gradient gel electrophoresis, a classical method complementary to ultracentrifugation, was employed to characterize lipoproteins (Blom et al., 2003). As showed here, BM lipoprotein‐like particles exhibited size and distribution comparable to those of adult serum lipoproteins.

Future research should explore how maternal lifestyle factors, particularly dietary modifications, influence the lipoprotein composition of BM. Such findings could guide public health initiatives to optimize breastfeeding quality and promote maternal metabolic health.

This study has certain limitations, including variation in the lactational age of the BM samples and the absence of detailed infant nutritional data, both of which could affect the lipoprotein and lipoprotein‐like composition of the analysed samples. Nevertheless, these results provide new evidence that human BM contains macromolecular structures with characteristics similar to plasma lipoproteins.

### 4.1 Conclusion

For the first time, the presence of lipoproteins in BM was characterized, providing new information regarding BM complexity.

## AUTHOR CONTRIBUTIONS

Gabriela Arenas, Andrea Morales, and Susana Contreras‐Duarte performed the main experiments; Susana Contreras‐Duarte wrote the main manuscript, and Andrea Leiva reviewed the main manuscript. All the authors approved the final version of the manuscript, agreed to be accountable for all aspects of the work in ensuring that questions related to the accuracy or integrity of any part of the work are appropriately investigated and resolved, and all persons designated as authors qualify for authorship, and all those who qualify for authorship are listed.

## CONFLICT OF INTEREST

The authors declare no conflicts of interest.

## Data Availability

All the data used for this manuscript will be available upon request. If this is the case, please contact Dr Susana Contreras at susana.contreras@uss.cl.
